# Type 2 deiodinase is expressed in anaplastic thyroid carcinoma and its inhibition causes cell senescence

**DOI:** 10.1530/ERC-23-0016

**Published:** 2023-04-13

**Authors:** Maria Angela De Stefano, Tommaso Porcelli, Raffaele Ambrosio, Cristina Luongo, Maddalena Raia, Martin Schlumberger, Domenico Salvatore

**Affiliations:** 1 Department of Public Health, University of Naples ’Federico II’, Naples, Italy; 2 IRCCS SDN, Naples, Italy; 3 Department of Clinical Medicine and Surgery, University of Naples ’Federico II’, Naples, Italy; 4 CEINGE Biotecnologie Avanzate Scarl, Naples, Italy; 5 Department of Endocrine Oncology, Gustave Roussy and University Paris-Saclay, Villejuif, France

**Keywords:** thyroid cancer, anaplastic thyroid cancer, deiodinases, thyroid hormone, cell senescence, p53

## Abstract

Anaplastic thyroid cancer (ATC) is a rare thyroid tumor that frequently originates from the dedifferentiation of a well-differentiated papillary or follicular thyroid cancer. Type 2 deiodinase (D2), responsible for the activation of the thyroid hormone thyroxine into tri-iodothyronine (T_3_), is expressed in normal thyroid cells and its expression is strongly downregulated in papillary thyroid cancer. In skin cancer, D2 has been associated with cancer progression, dedifferentiation, and epithelial–mesenchymal transition. Here, we show that D2 is highly expressed in anaplastic compared to papillary thyroid cancer cell lines and that D2-derived T_3_ is required for ATC cell proliferation. D2 inhibition is associated with G1 growth arrest and induction of cell senescence, together with reduced cell migration and invasive potential. Finally, we found that mutated p53^72R(R248W)^, frequently found in ATC, is able to induce D2 expression in transfected papillary thyroid cancer cells. Our results show that the action of D2 is crucial for ATC proliferation and invasiveness, providing a potential new therapeutic target for the treatment of ATC.

## Introduction

Anaplastic thyroid cancer (ATC) is a rare, aggressive tumor that accounts for only 1% of all thyroid cancers, but it is responsible for about one-third of all deaths from thyroid cancer (Fagin & Wells 2016). ATC originates from follicular thyroid cells and occurs at the end of a dedifferentiation process that frequently starts from differentiated cancer – either papillary (PTC) or follicular thyroid cancer – progresses through poorly differentiated thyroid cancer and then to ATC. In fact, ATC is commonly found in coexistence with more differentiated cancer cells, with which ATC cells share common driver mutations such as *BRAF^V600E^* or *RAS* (Nikiforov 2004, Cancer Genome Atlas Research 2014). ATC presents multiple additional somatic alterations that include mutations in *TP53*, *TERT* promoter, PI3K/AKT/mTOR pathway effector genes (Wang *et al.* 2022). The initial driver mutations of thyroid cancer facilitate tumor growth by increasing the transcriptional output of the mitogen-activated protein kinase (MAPK) signaling pathway that is paralleled by downregulation of several thyroid-specific gene expressions. In this context, an early event in thyroid tumorigenesis observed in PTC is the downregulation of type 1 (D1) and type 2 deiodinase (D2) genes, paralleled by the overexpression of type 3 deiodinase (D3) gene (Arnaldi *et al.* 2005, Romitti *et al.* 2012).

Deiodinases are a family of three seleno-enzymes that regulate the intracellular and extracellular levels of thyroid hormones (TH). D1 and D2 activate the pro-hormone thyroxine (T_4_) into the active tri-iodothyronine (T_3_) and are both expressed in normal thyroid cells, in which they contribute to thyroidal T_3_ production. Conversely, the D3 inactivates T_4_ and T_3_ into reverse T_3_ (rT_3_) and diiodothyronine (T2), respectively, and is not expressed in normal thyroid tissue (Bianco *et al.* 2002). Notably, the upregulation of D3 observed in PTCs is also observed in several other cancers, in which D3 acts to attenuate intracellular TH signaling, thereby promoting cell proliferation and cancer progression (Dentice *et al.* 2012, Di Girolamo *et al.* 2016, Miro *et al.* 2017).

The intracellular TH concentrations in late-stage thyroid cancer (i.e. ATC) are currently unknown. To investigate the intracellular TH metabolism in ATC, we first analyzed the expression of deiodinases in ATC and found that ATC cells express higher levels of D2 than PTC cells and similar to nontumoral thyroid cells, while significantly downregulate both D1 and D3 expression. In ATC cell lines, we found that D2 activity is crucial for cell proliferation since its blockade reduces ATC cell growth and induces cell senescence. Moreover, D2-blocking significantly affects the invasiveness and migration potential of ATC cells. Overall, these results show a previously unexplored role of D2 in ATC that opens a field for further investigations and potential new treatment targets.

## Materials and methods

### Cell lines and treatments

Human PTC cell lines (K1 and TPC-1) were gently donated by Dr F. Carlomagno (University of Naples ‘Federico II’). Human ATC cell line (8505c) and the immortalized human primary thyroid follicular epithelial cell line (Nthy-ori 3-1) were donated by Dr G. De Vita (University of Naples ‘Federico II’). KMH2 cells were purchased from Bioresources Cell Bank (JCRB; Osaka, Japan). TPC-1 and K1 cells present the CCDC6-RET gene fusion and the *BRAF^V600E^* mutation, respectively, while 8505c and KMH2 cells present mutations in *BRAF^V600E^*/*TP53* and* TERT* promoter, respectively. Nthy-ori 3-1, 8505c, and TPC-1 cells were cultured in Dulbecco’s modified Eagle’s medium (DMEM); KMH-2 cells were cultured in a 1:1 mixture of DMEM and RPMI 1640 medium; K1 cells were cultured in a 2:1:1 mixture of DMEM, Ham′s F12, and MCDB 105 medium. All media were supplemented with 10% fetal bovine serum, 1% penicillin/streptomycin, and 1% l-glutamine. Cells were incubated at 37°C in a 5% CO_2_ humidified incubator.

Cells were treated with 30 nM reverse-T_3_ (Sigma Aldrich #T0281) or 10 µM Forskolin (FSK, Calbiochem) for different amounts of time as indicated.

### Cell transfections

Cells were transfected using Lipofectamine 2000 (Thermo Fisher Scientific) according to the manufacturer’s instructions. Nthy-ori 3-1, 8505c, and KMH2 were co-transfected with reporter Luc plasmids Jun-Luc (Luongo *et al.* 2014) and CMV-Renilla. Luc activity was measured 48 h after transfection with the Dual Luciferase Reporter Assay System (Promega), and differences in transfection efficiency were normalized relative to the level of Renilla Luciferase. K1 cells were transfected with mutant P53 plasmid (pcDNA3 p53^72R(R248W)^, kindly provided by Dr C. Missero (University of Naples ‘Federico II’) or the corresponding empty vector. Each construct was transfected in triplicates in at least three separate transfection experiments. Luc/Renilla ratios are showed as mean ± s.e.m.

### Cell growth assay

Cells were seeded into six-well tissue culture dishes at a density of 6 × 10^3^ per well. Every 3 days, through 9 days of culture, three wells of the six-well dishes were trypsinized, and a cell count was performed using a Burker chamber under light microscopy.

### Quantitative PCR

Total RNAs were extracted with TRIzol reagent (Life Technologies) according to the manufacturer’s instructions and then reverse‐transcribed into cDNA by using LunaScript reverse transcriptase (New England BioLabs Inc., Ipswich, MA, USA) according to the manufacturer's instructions. Quantitative real-time PCR was performed using iQ5 Multicolor Real Time Detector System (BioRad Laboratories) with the fluorescent double-stranded DNA-binding dye SYBR Green (Applied Biosystems). Cyclophilin A gene served as the housekeeping gene controls for ΔC_T_ calculations [ΔC_T_ = (C_T_ of the target gene) − (C_T_ of housekeeping genes)]. Fold expression values were calculated using the 2^−ΔΔCT^ method, where ΔΔC_T_ = (ΔC_T_ of the treatment sample) − (ΔC_T_ of control samples) (with the control value normalized to 1). Three technical replicates were performed for all qPCR experiments.

### Western blot analysis

Total protein extracts from cells were run on a 15% sulfate-polyacrylamide electrophoresis gel and transferred to an Immobilon-P transfer membrane (Millipore). The membrane was then blocked with 5% BSA in phosphate-buffered saline, probed overnight at 4°C with appropriate antibodies anti-p16-INK4a (1:500, Invitrogen), anti-p27 Kip1/CDKN1B (1:500, Santa Cruz), anti- γ-H2A.X (1:4000, Abcam), anti-p21 WAF1/CIP1 (1:500, Cell Signaling DGS-60), and PARP (1:1000, Cell Signaling) washed and incubated with horseradish peroxidase-conjugated anti-mouse or anti-rabbit immunoglobulin G secondary antibody (1:3000). The membrane was incubated with anti-α-tubulin antibodies (1:1000, Santa Cruz) as a loading control. Western blots were run in triplicate. Antibody-labeled protein bands were revealed by using the Immobilon Western Chemiluminescent HRP Substrate (Millipore, WBKLS0500). The membrane images were analyzed using Image Lab version 5.2.1 (Biorad Laboratories) software.

### Flow cytometry

Cell cycle progression and cell death were evaluated by fluorescence-activated cell sorting (FACS) (BD FACS Canto II, Becton Dickinson, Franklin Lakes, NJ, USA) staining. Briefly, for cell cycle, the cells were fixed in ice-cold 70% ethanol and incubated on ice for 15 min. Subsequently, at least 10,000 single-cell events were recorded using FACS after staining with propidium iodide (PI) solution (PI 50 µg/mL and RNase A 200 µg/mL, Sigma Aldrich). G1, S, and G2/M fractions were quantified using MODFIT Lt3.0 Software. For cell death analysis, cells were incubated with annexin V-FITC (Molecular Probes, A13201) staining solution and PI staining solution (PI 2 μg/mL) for 15 min and analyzed by FACS. Data were analyzed with the MODFIT Lt3.0 Software.

### Ethynyl deoxyuridine incorporation assay

A 5-ethynyl-2′-deoxyuridine (EdU) assay was detected using the Click-It kit (Invitrogen, C10337) according to the manufacturer’s instructions. Briefly, cells were fixed after incubation with 10 µM EdU labeling solution for 2 h. Subsequently, EdU was detected using the Click-It kit. Data on EdU incorporation are reported as the percentage of EdU^+^ vs total cells (measured by DAPI). Images were acquired with an IX51 Olympus inverted fluorescent microscope.

### *In vitro* colony formation assay

The colony formation assay was adapted to the 12-well culture plates and assayed for three-dimensional growth of tumor cells in Matrigel. Growth factor reduced Matrigel (BD Biosciences, Bedford, MA, USA) from frozen stock was kept overnight at 4°C. Once liquified, Matrigel was added to a 12-well culture plate (100 μL/well) and allowed to solidify by incubating at 37°C for at least 20 min. Appropriate culture medium was added (400 μL/well) on top of the Matrigel followed by cells (5000 cells/well). The plates were then incubated in a humidified CO_2_ incubator at 37°C for 2 weeks.

### Senescence-associated β-galactosidase staining

Senescence was determined by measuring the senescence-associated β-galactosidase (SA-β-Gal) activity. Briefly, cells were fixed for 3–5 min at room temperature in 3% formaldehyde and incubated with fresh SA-β-Gal staining at 37°C for 3 h. The staining solution included 1 mg/mL of 5-bromo-4-chloro-3-indolyl β-d-galactopyranoside (B4252, Sigma-Aldrich), 40 mM citric acid/sodium phosphate (pH 6.0), 5 mM potassium ferrocyanide (P3289, Sigma), 5 mM potassium ferricyanide (P4066, Sigma), 150 mM NaCl (S9888, Sigma), and 2 mM MgCl_2_ (M9272, Sigma). The percentage of senescent cells was calculated by the number of β-galactosidase positive cells (blue) out of a total number of cells per field. Images were acquired with an IX51 Olympus inverted fluorescent microscope.

### Migration assay

Migration ability was assessed by wound healing assay. Cells were plated in p60 culture plates and grown to confluence, and then treated with mitomycin (M4287, Sigma-Aldrich) to inhibit cell proliferation. A scratch was generated on a confluent cell monolayer with a 200 µL tip pipette. The closing of the wound was monitored by capturing images at different time points using a phase-contrast microscope and the percentage of remaining wounds was measured using Cell^a^ software (Olympus Biosystem GmbH, London, UK).

### Transwell invasion assay

Invasion ability was assessed by Matrigel matrix assay. Briefly, cells (30 × 10^4^ cells/well) were treated with mitomycin (Sigma-Aldrich M4287) to inhibit cell proliferation and seeded into upper transwell chambers precoated with Matrigel matrix. After incubation for 48 h at 37°C, the cells that had invaded through the chamber membrane were fixed with paraformaldehyde 4% for 15 min at room temperature and stained with 1% crystal violet solution for 10 min. Cells were observed under a light microscope, the images were acquired, and the relative number of cells was calculated on the total area using the ImageJ software.

### Statistical analysis

Significant differences were calculated using ANOVA, and multiple *t* tests with *P* < 0.05 were considered as statistically significant. All statistics and graphics were performed using GraphPad Prism9. In all figures, error bars represent the s.e.m. A value of *P* < 0.05 was considered significant (*P < 0.05; **P < 0.01; ***P < 0.001).

## Results

### Type 2 deiodinase and TH signaling genes are upregulated in ATC vs PTC cells

**Figure 1:**
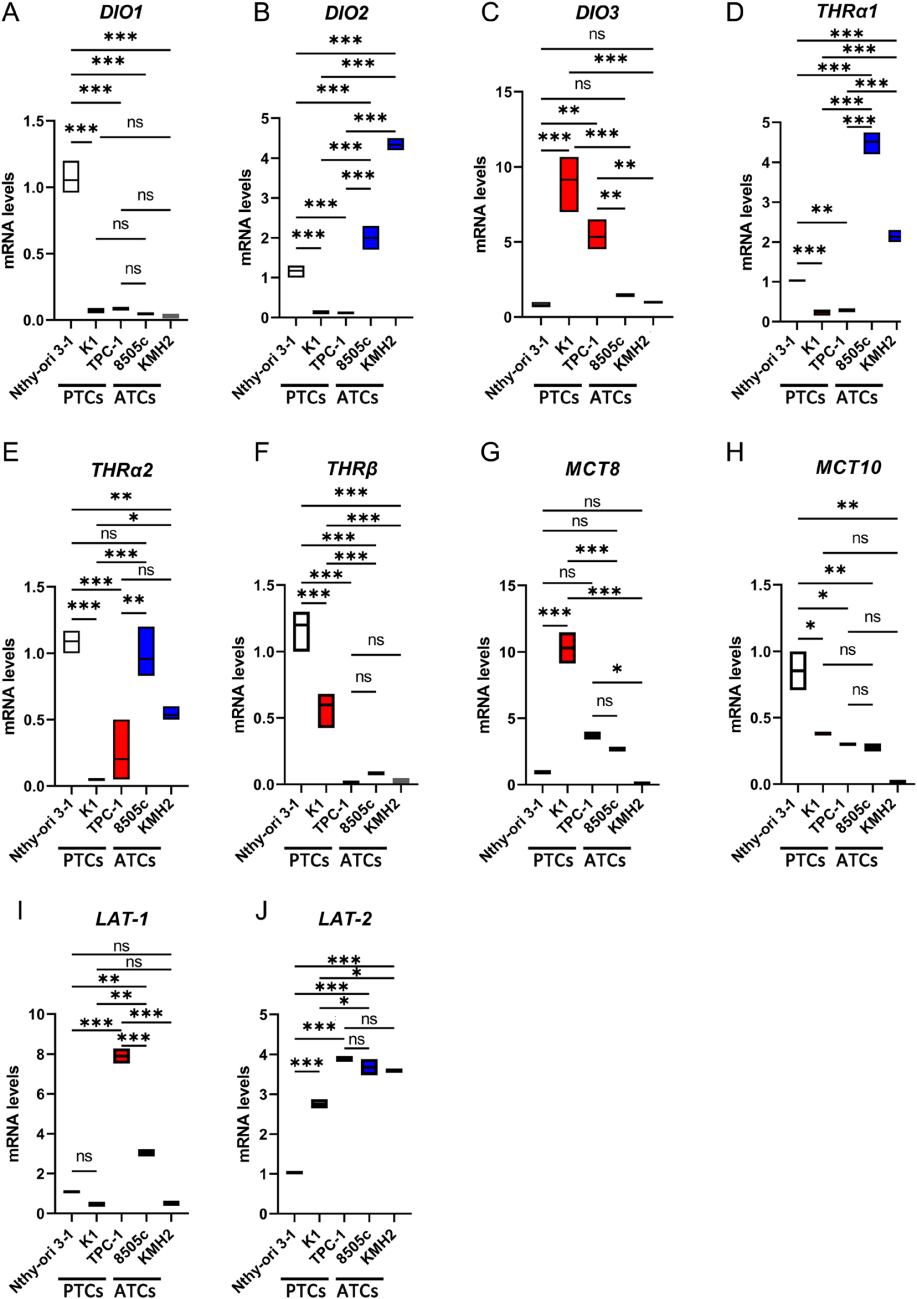
Expression analysis by mRNA levels of TH-metabolism-related genes in nontumoral thyroid cells compared to PTC and ATC cells. (A) *DIO1* is markedly downregulated in both PTC (K1 and TPC-1) and ATC (8505c and KMH2) cells. (B–C) *DIO2* (B) is significantly upregulated in ATC cells compared to Nthy-ori and PTC, while PTC cells significantly upregulated *DIO3* (C) compared to Nthy-ori and ATC. (D–F) Thyroid hormone receptors *THR*α1 (D), *THR*α2 (E), *THR*β, and (F) mRNA levels in nontumoral thyroid cells compared to PTC and ATC cells. (G–J) Thyroid hormone transporters *MCT8* (G), *MCT10* (H), *LAT-1* (I), and *LAT-2* (J) mRNA levels in nontumoral thyroid cells compared to PTC and ATC cells. mRNA levels were measured by qRT-PCR. Data are expressed as mean ± s.e.m. of at least three independent experiments. * *P* < 0.05, **P < 0.01, ***P < 0.001.

To shed light on the TH intracellular metabolism in late stages of thyroid tumorigenesis, we analyzed the mRNA expression of TH transporters, deiodinases, and TH nuclear receptors in two ATC cell lines, namely, 8505c and KMH2. Results were compared to nontumoral thyroid cells Nthy-ori 3-1 and to two PTC cell lines, namely, K1 and TPC-1. While D1 was strongly downregulated in both PTC and ATC cells compared to Nthy-ori 3-1 (Fig. 1A), both D2 and D3 showed an opposite expression profile between PTC and ATC. Strikingly, both 8505c and KMH2 significantly upregulated D2 expression, while PTC cells showed a marked D2 downregulation (Fig. 1B). While both K1 and TPC-1 cells strongly overexpressed D3 compared to Nthy-ori 3-1, D3 was not detectable in ATC cells (Fig. 1C). The elevated D3 expression in PTC cells, leading to a reduction in intracellular T_3_ concentration, was consistent with the downregulation of TH nuclear receptors in both K1 and TPC-1 compared to nontumoral thyroid cells (Fig. 1D, E and F). In contrast, in ATC cells, a significantly increased expression of the thyroid hormone nuclear receptor α1 isoforms (THRα1) was observed compared to nontumoral thyroid cells, while the expression of THRβ – expressed in nontumoral thyroid cells – and THRα1 was significantly downregulated (Fig. 1D, E and F). The expression of various TH transporters was variable in different thyroid cancer cell lines (Fig. 1G, H, I and J). In particular, *MCT10* was minimally expressed in all thyroid cancer cell lines tested (Fig. 1G and H), while *LAT2* was overexpressed in cancers compared to nontumoral control cells (Fig. 1I and J). Overall, these results indicate that in ATC cells D2 is highly expressed together with a specific fingerprint of active TH signaling machinery.

### The inhibition of D2 decreases ATC cell proliferation

**Figure 2:**
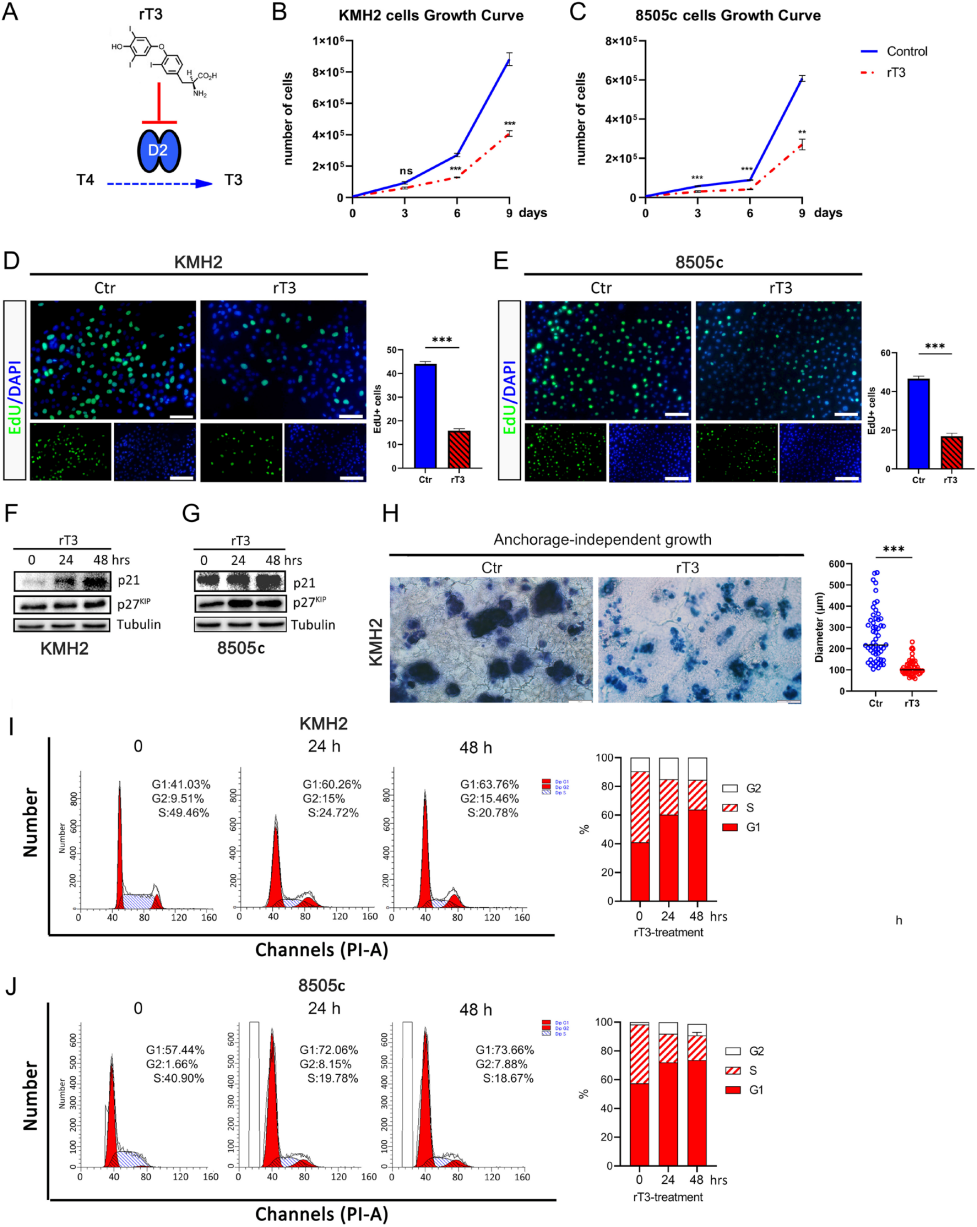
The inhibition of D2 negatively affects ATC cell proliferation. (A) Representation of D2-blocking by rT3. (B–C) Growth curves of control and rT_3_-treated KMH2 (B) and 8505c (C) cells. (D) Representative immunofluorescence of EdU (green) and DAPI (blue) in controls and rT_3_-treated KMH2 cells (left, scale bar 100 µm) and quantification of percentage of EdU-positive cells (right). (E) Representative immunofluorescence of EdU (green) and DAPI (blue) in controls and rT_3_-treated 8505c cells (left, scale bar 100 µm) and quantification of percentage of EdU-positive cells (right). (F–G) Protein levels by Western blot of cell cycle inhibitors p21 and p27KIP in controls and rT_3_-treated KMH2 cells (F) and 8505c (G) cells. Tubulin served as a loading control. (H) Anchorage-independent growth of KMH2 cells treated with rT_3_ evaluated by soft matrigel assay. Cell colony diameters were examined 2 weeks after plating (scale bar 100 µm). The experiment was performed in triplicate. (I–J) Cell cycle distribution measured in asynchronized KMH2 (I) and 8505c (J) cells at T0, T1 (24 h), and T2 (48 h) by flow cytometry using propidium iodide staining. The percent of cells in each cell cycle phase is shown in the bar graph (right). Data are expressed as mean ± s.e.m. of at least three independent experiments. *P < 0.05, **P < 0.01, ***P < 0.001.

To determine whether D2 plays any role in ATC cells, we treated KMH2 and 8505c withrT_3_ – a potent and specific D2 inhibitor (Bianco *et al.* 2002). (Fig. 2A) – and a significant reduction in cell proliferation was observed in both ATC cell lines compared to untreated controls (Fig. 2B and C). Accordingly, EdU incorporation was significantly reduced at 48 h after rT_3_ treatment compared to controls (Fig. 2D and E), and cell cycle inhibitors p21 and p27^KIP^ showed a significant upregulation in rT_3_-treated ATC cells (Fig. 2F and 2G). Moreover, we found that also the anchorage-independent growth of ATC cells was affected by D2-inhibition, as shown by the significantly reduced diameter of rT_3_-treated cell clones in soft matrigel, compared to untreated controls (Fig. 2H).

To get insight into the growth reduction of D2-depleted ATC cells, we examined the cell cycle phase distribution by flow cytometry in asynchronized ATC cells upon rT_3_ treatment. Significantly, 24 and 48 h after treatment with rT_3_, both KMH2 and 8505c cells showed a significant shift toward the G1 phase of the cell cycle, while the population in S-phase was reduced by about 50% (Fig. 2I and J).

### D2-blocking induces cell senescence

**Figure 3:**
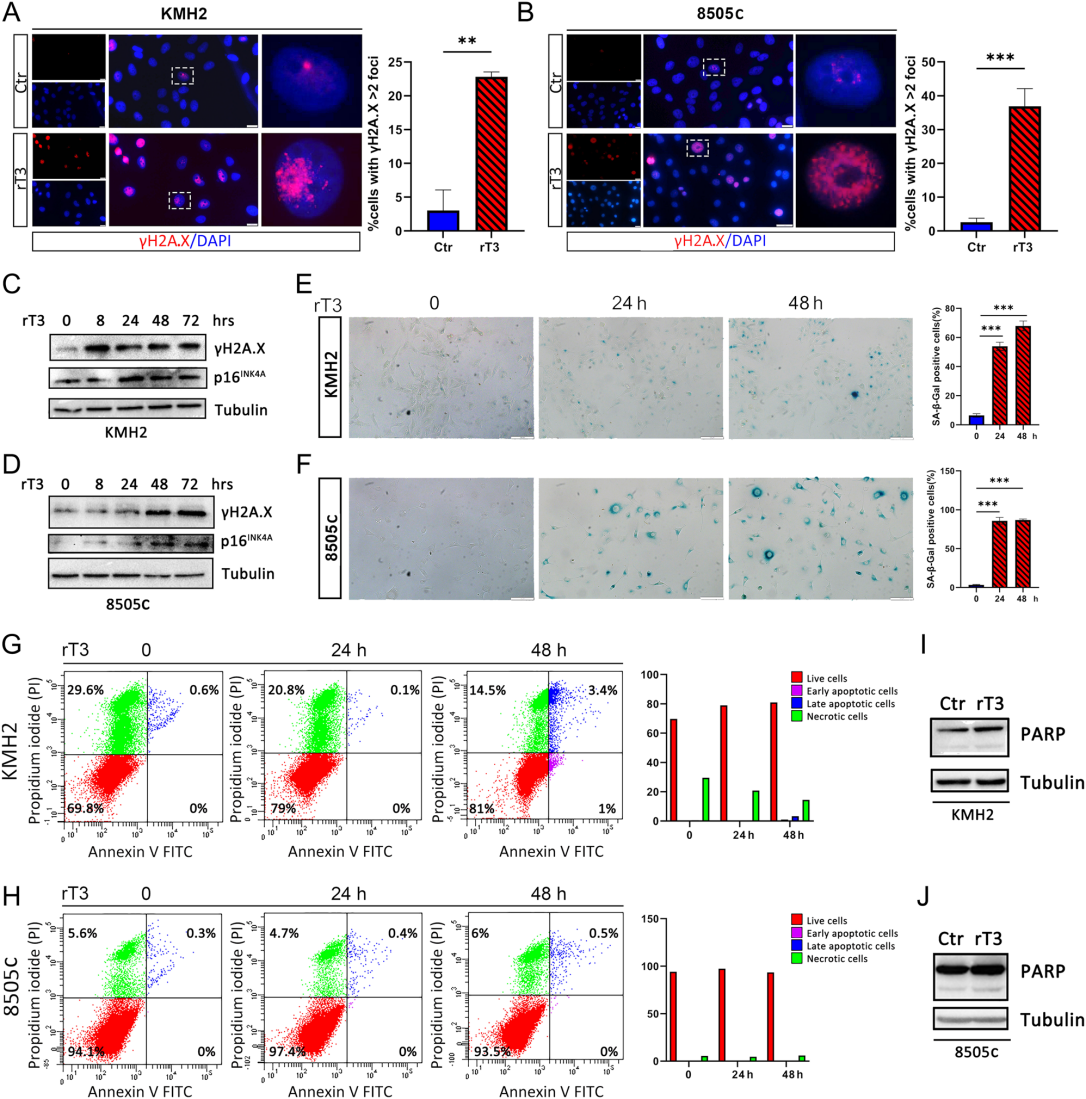
D2 inhibition induces senescence of ATC cells. (A–B) Expression of senescence marker γ-H2A.X. Immunofluorescence staining of γ-H2A.X in controls and rT_3_-treated KMH2 (A) and 8505c (B) cells (scale bar 20 µm) and quantification of the percentage of cells with γ-H2A.X > 2 loci (right). (C–D) Protein levels of γ-H2A.X and p16^INK^ (senescence markers) in KMH2 (C) and 8505c (D) cells at 0, 8, 24, 48, and 72 h upon rT_3_ treatment. (E–F) Images of SA-β-Gal staining in KMH2 (E) and 8505c (F) cells at 0, 24, and 48 h from rT_3_ treatment (left, scale bar 100 µm) and quantification of the percentage of SA-β-Gal positive cells referred to the total amount of cells on the plate (right). (G–H) Flow cytometry detection of phosphatidylserine exposure analyzed with annexin V and propidium iodide in KMH2 (G) and 8505c (H) cells (left) and quantification (right). (I–J) Protein levels by Western blot of PARP in KMH2 (I) and 8505c (J) controls compared to rT_3_-treated cells. Tubulin served as a loading control. Data are expressed as mean ± s.e.m. of at least three independent experiments. * *P* < 0.05, **P < 0.01, ***P < 0.001.

The increase in ATC cells that entered the G1-phase upon D2-inhibition prompted us to analyze whether the growth reduction consequent to the D2-blocking was associated with cell senescence. In both ATC cell lines, D2-blocking significantly increased the percentage of cells with γ-H2A.X foci > 2 (i.e. an established marker of senescence-associated DNA damage) (Fig. 3A and B). This was confirmed by Western blot analysis for γ-H2A.X and p16^INK4A^ levels, both significantly increased after treatment with rT_3_ (Fig. 3C and D). To verify the specificity for ATC cells of the described effects of D2-blocking, we treated Nthy-ori 3-1 and TPC-1 cells (nontumoral thyroid and PTC cells, respectively) with rT_3_ and in a time-course experiment, rT_3_ induced no change in γ-H2A.X protein levels by Western blot analysis (Supplementary Fig. 1, see section on supplementary materials given at the end of this article).

To confirm that D2-blocking induced senescence of ATC cells, KMH2 and 8505c cells were analyzed for SA-β-Gal. Reverse T_3_-treated ATC cells showed a significantly higher number of SA-β-Gal positive cells compared to controls, confirming that D2-inhibition strongly induced senescence (Fig. 3E and F). To exclude that the reduction in growth rate associated with the D2-inhibition was also due to induced apoptosis, we measured the annexin V/PI staining by FACS analysis in ATC cells 24 and 48 h after treatment with rT_3_. We found that the percentage of apoptotic cells was unaffected by rT_3_ treatment (Fig. 3G and H). Accordingly, cell apoptosis measured by Western blot analysis for PARP cleavage showed no differences between rT_3_-treated and untreated ATC cells (Fig. 3I and J).

### Migration and invasiveness potential of ATC cells are hampered by D2-inhibition

**Figure 4:**
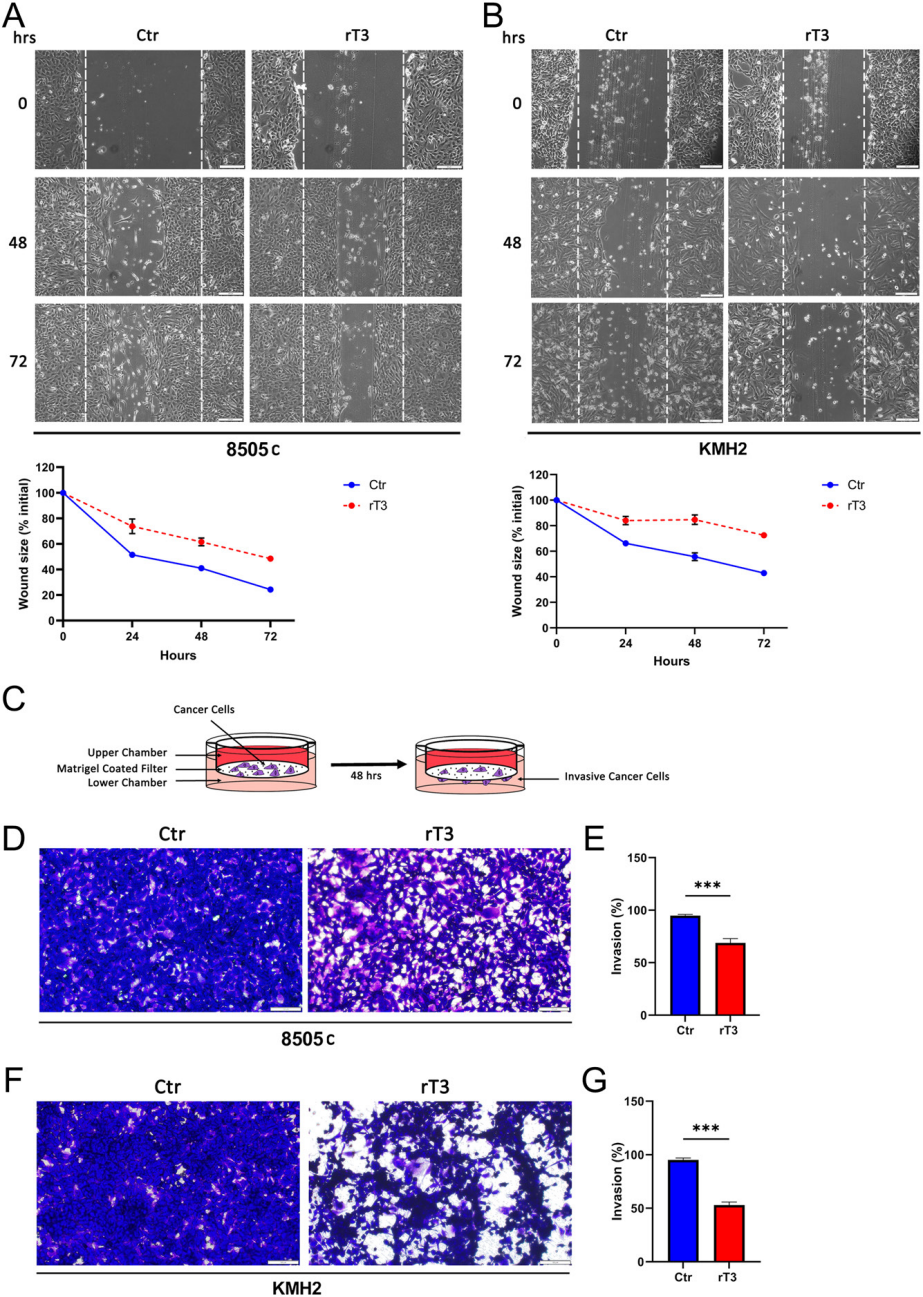
Consequences of D2-blocking on migration and invasiveness potential of ATC cells. (A–B) Upper: Images of wound healing scratch of 8505c (A) and KMH2 (B) 0, 48, and 72 h after treatment with rT_3_ vs untreated controls (scale bar represents 200 µm). Below: percentage of unhealed wound. (C) Schematic image of invasion assay. (D–G) Images of matrigel-coated membrane chamber assay for the invasive potential of rT_3_-treated 8505c (D) and KMH2 cells (F) vs controls (scale bar represents 200 µm). Percentage of rT_3_-treated 8505c (E) and KMH2 cells (G) that have invaded matrigel vs controls. Data are expressed as mean ± s.e.m. of at least three independent experiments. *P < 0.05, **P < 0.01, ***P < 0.001.

Next, we tested whether D2 action influences the migration and invasiveness potential of ATC cells. First, we analyzed cell migration through a wound healing experiment. We found that the migration ability was significantly reduced (by about 40%) in rT_3_-treated ATC cells compared to untreated controls (Fig. 4A and B). We then analyzed the ATC invasiveness potential by using the matrigel-coated membrane chamber assay (Fig. 4C). We observed a reduced invasive potential for rT_3_-treated ATC cells (of about 35% and 50% in 8505c and KMH2 cells, respectively) compared to controls (Fig. 4D, E, F and G).

### Potential signaling pathways sustaining D2 expression in ATC cells

**Figure 5:**
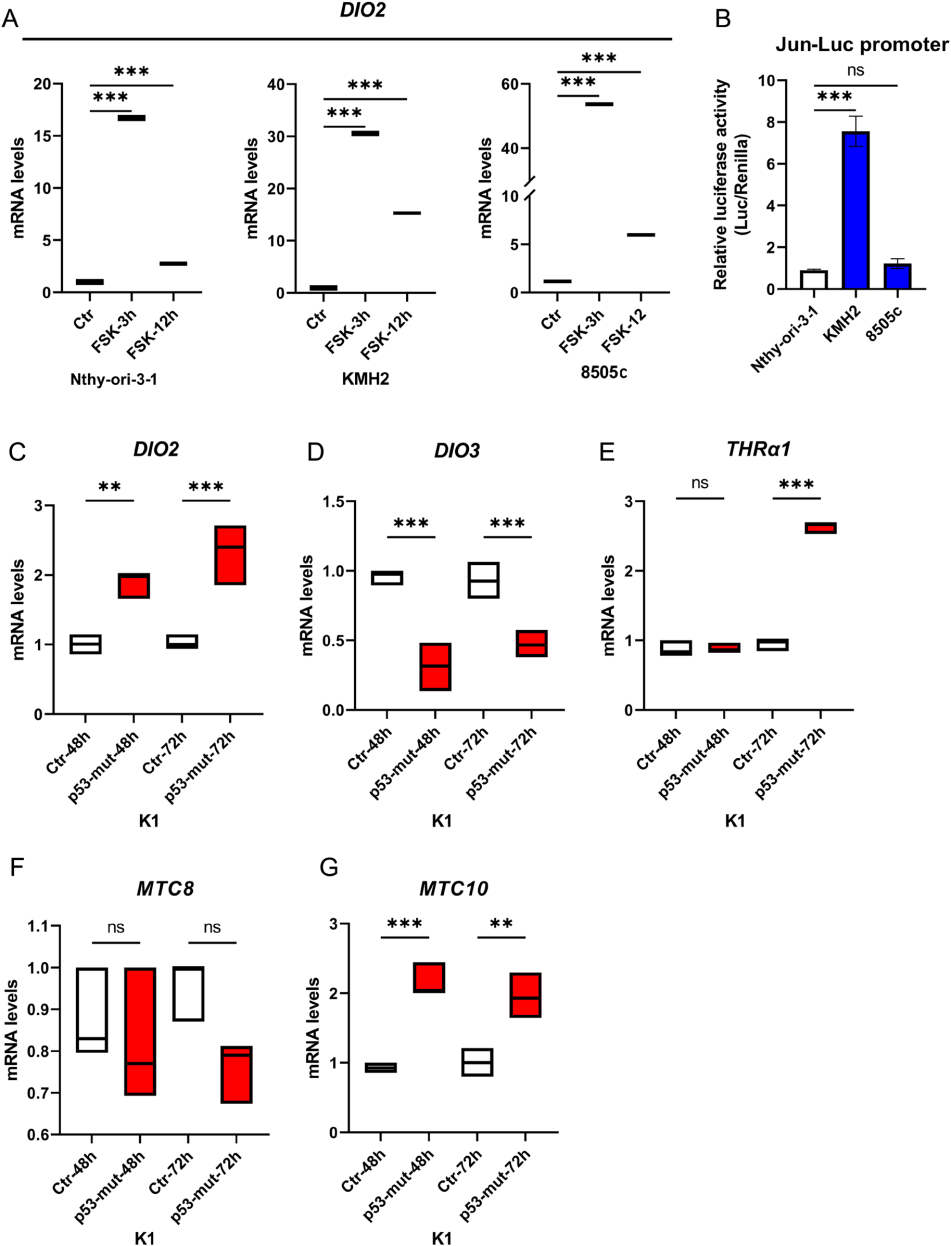
Dio2 expression in thyroid cancer is sensitive to cAMP and mutant p53 signaling. (A) Treatment with 10 µM of forskolin (FSK) for 3 and 12 h:* DIO2* mRNA levels were measured by RT-PCR in Nthy-ori 3-1, 8505c, and KMH2 cell lines. (B) Jun-Luc promoter (a cAMP-responsive promoter) was transfected to evaluate the basal cAMP activity in Nthy-ori 3-1, 8505c, and KMH2 cell lines. (C-G) Transfection of mutant p53^72R(R248W)^ in PTC (K1) cells:* DIO2* (C), *DIO3* (D) *THRα1* (E),* MCT8* (F), and* MCT10* (G) mRNA expression at 24 and 72 h. Data are expressed as mean ± s.e.m. of at least three independent experiments. *P < 0.05, **P < 0.01, ***P < 0.001.

To investigate potential inducers of D2 in ATC, we wondered whether the cAMP, a well-known marker of thyroid cell differentiation and a prominent transcriptional D2 inducer in normal thyroid cells, was still active in the ATC cellular context. ATC cells were treated with 10 µM forskolin (FSK), a potent adenylate cyclase activator (Robbins *et al.* 1996) for 3 and 12 h. The expression levels of D2 strongly increased in ATC cells to a similar extent to what was observed in nontumoral thyroid cells (Nthy-ori 3-1) upon FSK treatment (Fig. 5A), suggesting that the control by cAMP signaling of the D2 gene transcription is preserved in ATC cells. Interestingly, the basal cAMP signaling levels measured in ATC cells are significantly elevated in KMH2 cells compared to nontumoral thyroid cells, suggesting that cAMP may be involved in D2 upregulation in the ATC cells (Fig. 5B).

Mutation in p53, a frequent genetic event in human ATC, is often associated with progression from a differentiated to an undifferentiated (i.e. anaplastic) thyroid cancer phenotype. Therefore, we asked whether D2 expression, absent in PTC cells, could be induced in this cell context by a mutant p53. To this aim, we transfected a mutant p53 isoform (p53^72R(R248W)^) in the PTC cell line K1. Strikingly, mutant p53 markedly upregulated D2 and downregulated D3 compared to controls (Fig. 5C and 5D). Importantly, mutant p53 also increased the expression of the TRα1 TH receptor and MCT10 (not MCT8) TH transporter, similar to the expression profile of ATC cell lines, indicating that a global transition in TH signaling can be induced by a mutated p53 in a PTC cellular context (Fig. 5E, F and G).

## Discussion

In several cancer settings, a change in deiodinase expression profile occurs when compared to the normal cell condition, and this is thought to customize the intracellular TH levels to promote cancer growth (Nappi *et al.* 2021). This is also the case of differentiated thyroid cancer, in which in the early phases of tumorigenesis, that is, in PTC, D3 is overexpressed to achieve a hypothyroid nuclear environment possibly promoting the minimal growth of these tumors (Romitti *et al.* 2016). In the present study, we aimed to investigate whether a change in deiodinase expression also occurs in late-stage thyroid cancer, that is, in ATC, and whether the manipulation of intracellular T_3_ concentration could alter the biological behavior of ATC cells. Our results show that in ATC cells the TH signaling is markedly increased compared to PTC cells and that this increase is sustained by the activity of D2. This change is relevant to sustain the cancer phenotype, and the inhibition of D2 dramatically altered the viability of ATC cells, leading to cell senescence and blunted tumorigenic potential.

The reported opposite regulation of D3 and D2 between early and late-stage thyroid cancers is in line with reports from other cancers. In a mouse model of skin squamous cell carcinoma, we previously demonstrated that tumor cells overexpress D3 to enhance tumor growth in the initial steps of tumorigenesis, while upregulates D2 in later phases to progress to a higher grade of disease (Miro *et al.* 2019). Similarly, we found that D3 expression levels in colorectal carcinomas were negatively correlated with the histological grade of the disease (Dentice *et al.* 2012). Also, a study on 59 colorectal carcinomas reported a significant overexpression of THRα1 in advanced-stage cancers compared to early-stage cancers (Uchuya-Castillo *et al.* 2018), similar to what we observed in ATC cells, that markedly upregulate THRα compared to nontumoral thyroid cells while inhibiting the THRβ transcription.

Despite multiple evidence of alterations in TH signaling in cancer contexts, the functional role of TH in cancer development, maintenance, and progression is still mostly unknown. However, the modulation of deiodinase expression along cancer dedifferentiation supports the concept that TH action in cancer is highly time- and cell context-specific. The progression to ATC in thyroid tumorigenesis is associated with additional genetic mutations to the initial tumor driver mutation, which strongly increases the MAPK transcriptional output of ATC compared to PTC. As a consequence, a study of human ATC (Landa *et al.* 2016) showed that thyroid-specific genes such as *SLC5A5*, *TG*, *TPO,* and *DIO1* are significantly downregulated in ATC compared to PTC. Strikingly, *DIO2* – which is expressed in normal thyroid tissue – was significantly upregulated in ATC vs PTC (Cancer Genome Atlas Research 2014), suggesting that its expression in the context of ATC plays a different role than in normal thyroid cells, likely necessary for cancer progression. Indeed, we showed that D2-blocking – and the consequent reduction in intracellular T_3_ levels – in ATC cells is strongly associated with cell senescence. In other cancer contexts, such as colorectal cancer (Yang *et al.* 2021), an increase in proliferation and metastasis through non-genomic action of T_4_ by the binding to plasma membrane receptors, such as αvβ3 integrin, has been reported (Davis *et al.* 2016). In our study, D2-blocking may have potentially increased the intracellular T_4_ levels in ATC cells. However, the elevated T_4_ concentration in serum – in rapid equilibrium with the intracellular milieu – makes a putative D2-effect on T_4_ concentrations negligible.

The upstream regulator of D2 in the context of ATC is unknown. In normal thyroid cells, D2 is induced by cAMP, which directly binds to the D2 gene promoter region (Bartha *et al.* 2000). This signaling pathway has definitely a role in benign thyroid disease, such as in Graves-Basedow disease and in functional follicular neoplasms, where it is responsible for the increased T_3_ output from the thyroid gland (Dumont *et al.* 1992). Here, we showed that cAMP signaling is still present in the regulation of D2 in ATC, but its role remains unknown. However, other pathways may contribute to its upregulation, including mutant p53, that is strongly related to the PTC progression to ATC and that significantly increased D2 gene expression. However, several other D2 inducers may also be potentially involved. As an example, in a recent study in skin cancer, we showed that the NANOG transcription factor – involved in the self-renewal of embryonic stem cells and overexpressed in many cancers – promotes D2 transcription and is co-expressed with D2 in late-stage squamous cancers (Nappi *et al.* 2020).

In conclusion, this is the first study exploring the role of D2 in ATC in thyroid cancer and demonstrates that advanced thyroid cancer customizes intracellular metabolism, irrespectively to the plasma T_3_ concentrations. Our *in vitro* data on ATC cells suggest that D2 expression may be significantly related to thyroid cancer progression, but studies in mouse model of cancer progression are needed to confirm these data *in vivo*. Studies of deiodinase expression in human tumors are also needed and are in progress to confirm these observations.

## Supplementary Material

Supplementary Figure 1

## Declaration of interest

All authors declare no competing interests.

## Funding

This study was supported by AIRC Individual Grant to DS (IG 2022, Project Number 27729) and by National Center for Gene Therapy and Drugs based on RNA Technology MUR-CN3 CUP E63C22000940007 to DS.

## Author contribution statement

MADS, TP, RA, MR, and CL performed the *in vitro* and *in vivo* experiments. MADS prepared the figures. DS designed the overall study and contributed to experiment supervision. MADS, MS, and DS contributed to data interpretation. MADS and TP wrote the paper. All authors discussed the results and provided input on the manuscript.
