# Supplementary figures and images for: Type 2 deiodinase is expressed in anaplastic thyroid carcinoma and its inhibition causes cell senescence
Source: Endocr Relat Cancer. 2023 Apr 13;30(5):e230016. doi: 10.1530/ERC-23-0016 (PMC10160549; doi:10.1530/ERC-23-0016)

**A**

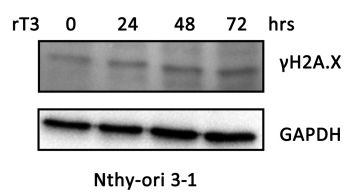

**B**

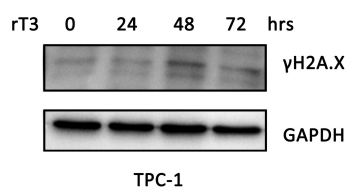

**S1**

Supplement: Supplementary Figure 1 [file supplementary_figure_1.pdf]
